# Teleost Fish-Specific Preferential Retention of Pigmentation Gene-Containing Families After Whole Genome Duplications in Vertebrates

**DOI:** 10.1534/g3.118.200201

**Published:** 2018-03-29

**Authors:** Thibault Lorin, Frédéric G. Brunet, Vincent Laudet, Jean-Nicolas Volff

**Affiliations:** * Institut de Génomique Fonctionnelle de Lyon, École Normale Supérieure de Lyon, UMR 5242 CNRS, Université Claude Bernard Lyon I, Université de Lyon, 46 Allée d’Italie, 69364 Lyon Cedex 07, France; † Observatoire Océanologique de Banyuls-sur-Mer, UMR CNRS 7232 BIOM; Sorbonne Université; 1, Avenue Pierre Fabre, 66650 Banyuls-sur-Mer, France

**Keywords:** pigmentation, chromatophores, vertebrates, teleost, whole-genome duplication

## Abstract

Vertebrate pigmentation is a highly diverse trait mainly determined by neural crest cell derivatives. It has been suggested that two rounds (1R/2R) of whole-genome duplications (WGDs) at the basis of vertebrates allowed changes in gene regulation associated with neural crest evolution. Subsequently, the teleost fish lineage experienced other WGDs, including the teleost-specific Ts3R before teleost radiation and the more recent Ss4R at the basis of salmonids. As the teleost lineage harbors the highest number of pigment cell types and pigmentation diversity in vertebrates, WGDs might have contributed to the evolution and diversification of the pigmentation gene repertoire in teleosts. We have compared the impact of the basal vertebrate 1R/2R duplications with that of the teleost-specific Ts3R and salmonid-specific Ss4R WGDs on 181 gene families containing genes involved in pigmentation. We show that pigmentation genes (PGs) have been globally more frequently retained as duplicates than other genes after Ts3R and Ss4R but not after the early 1R/2R. This is also true for non-pigmentary paralogs of PGs, suggesting that the function in pigmentation is not the sole key driver of gene retention after WGDs. On the long-term, specific categories of PGs have been repeatedly preferentially retained after ancient 1R/2R and Ts3R WGDs, possibly linked to the molecular nature of their proteins (*e.g.*, DNA binding transcriptional regulators) and their central position in protein-protein interaction networks. Taken together, our results support a major role of WGDs in the diversification of the pigmentation gene repertoire in the teleost lineage, with a possible link with the diversity of pigment cell lineages observed in these animals compared to other vertebrates.

Pigmentation is a highly diverse trait in the animal kingdom, and its spectacular and intriguing features have always been an object of interest and fascination both for naturalists and the general public. Back then, Alfred R. Wallace has been among the first in the 19^th^ century to understand pigmentation (called “coloration” at the time) in an adaptive context, and his classification of the different pigmentation functions remains largely accepted today (Wallace 1877; Caro 2017). What Wallace could not know at this time was the cellular and genetic bases of pigmentation, a topic that has been much studied since then, especially in vertebrates.

Except for structural colors such as in bird feathers, vertebrate pigmentation is mostly due to a single group of precursor cells that is a vertebrate-specific innovation: the neural crest cells (Green *et al.* 2015). While this transient group of cells emerging from the dorsal part of the neural tube has many derivatives during development (such as cranial cartilage, bone and connective tissue, adrenergic cells, sensory neurons), one category of neural crest-derived cells is of paramount importance in the context of pigmentation diversity: the pigment cells (Lapedriza *et al.* 2014). Among vertebrates, teleost fish harbor the highest number of pigment cell types. While mammals only have melanocytes, teleosts have more than eight different cells (sub)types described: black melanocytes, silvery iridophores and possibly also iridophore-like cells, orange-yellow xanthophores, red erythrophores, whitish leucophores, blue cyanophores, dichromatic erythro-iridophores and cyano-erythrophores, and fluorescent chromatophores (Bagnara and Hadley 1973; Fujii 1993; Goda and Fujii 1995; Goda *et al.* 2011, 2013; Schartl *et al.* 2016). New pigment cell types are still being described in teleosts (Wucherer and Michiels 2012; Goda 2017) (see Figure S1) and the already large list of genes known to affect pigmentation in vertebrates is constantly increasing (see for instance (Mallarino *et al.* 2016)). Most pigmentation genes characterized so far in vertebrates come from mouse mutant studies (Hoekstra 2006), but others have been identified more recently in birds and teleost fish including zebrafish and medaka, which are particularly useful species to study pigment cell types absent from mammals (Roulin and Ducrest 2013; Lopes *et al.* 2016; Mundy *et al.* 2016; Frohnhöfer *et al.* 2016; Nüsslein-Volhard and Singh 2017). Most melanin pigmentation genes found in mammals are also involved in pigmentation in teleosts, suggesting conservation of an ancestral melanin production pathway in vertebrates (Rawls *et al.* 2001; Braasch *et al.* 2008).

The evolutionary origin of the neural crest, which has sometimes being referred to as the “4th germ layer in vertebrates” (Gilbert 2000), is not clear. It has been suggested that the evolutionary emergence of the neural crest and the diversification of its cellular derivatives including pigment cells has required many changes in gene regulatory networks. This has been proposed to be linked to the occurrence of two rounds of whole-genome duplications (WGDs) called 1R/2R early during vertebrate evolution after their split from urochordates (Davidson 2006; Green *et al.* 2015). Such events of genome duplications are revealed by the presence of homeologous chromosomes containing sets of paralogous genes known as “ohnologs” (Wolfe 2001; Turunen *et al.* 2009), in reference to Susumu Ohno and his seminal work on the genome duplication hypothesis (Ohno 1970, 1999). During subsequent evolution, additional WGDs occurred independently in different vertebrate lineages (Session *et al.* 2016). At the basis of teleost fish, a third round of WGD has taken place ca. 300 million years ago (teleost-specific genome duplication or Ts3R) (Meyer and Schartl 1999; Woods *et al.* 2000; Jaillon *et al.* 2004; Brunet *et al.* 2006). Within teleosts, Ts3R was followed by more recent lineage-specific events including a fourth round of WGD that occurred in salmonids ca. 100 million years ago (Salmonid-specific genome duplication or Ss4R; (Macqueen and Johnston 2014; Berthelot *et al.* 2014; Lien *et al.* 2016)) and genome tetraploidization in cyprinids (Li *et al.* 2015). The teleost lineage is thus considered to be prone to WGDs, even if such events also occurred in non-teleost ray-finned fishes, *e.g.*, sturgeons (Havelka *et al.* 2013).

Our aim is to investigate how different events of genome duplications have contributed to the evolution and diversification of the pigmentation gene repertoire in teleost fish and other vertebrates. Indeed, many pigmentation genes have been duplicated in teleosts by the Ts3R WGD (Braasch *et al.* 2008, 2009a, 2009b). Previous studies only focused on the impact of Ts3R on the evolution of this pathway (Braasch *et al.* 2008, 2009a). Here, we have compared the impact on the pigmentation gene repertoire of the ancestral 1R/2R vertebrate duplications and the teleost Ts3R and Ss4R WGDs. We show that pigmentation genes and their paralogs, if any, have been more frequently retained as duplicates than other genes after the fish-specific Ts3R and Ss4R WGDs, but not after the early vertebrate 1R/2R, with a possible link with the diversification of pigment cell types specifically found in teleost fish.

## MATERIAL AND METHODS

### Pigmentation genes and their functional classification

Vertebrate *pigmentation genes* (PGs) are defined here as genes that are involved in the differentiation of a neural-crest derived pigment cell in at least one vertebrate species. This strict definition excludes in particular pigment cells present in the retinal pigmented epithelium, an anterior neural plate derivative (Ramón Martínez-Morales *et al.* 2004). According to this definition, one PG can be implicated in pigment cell differentiation in one species but not in others.

**Figure 1:**
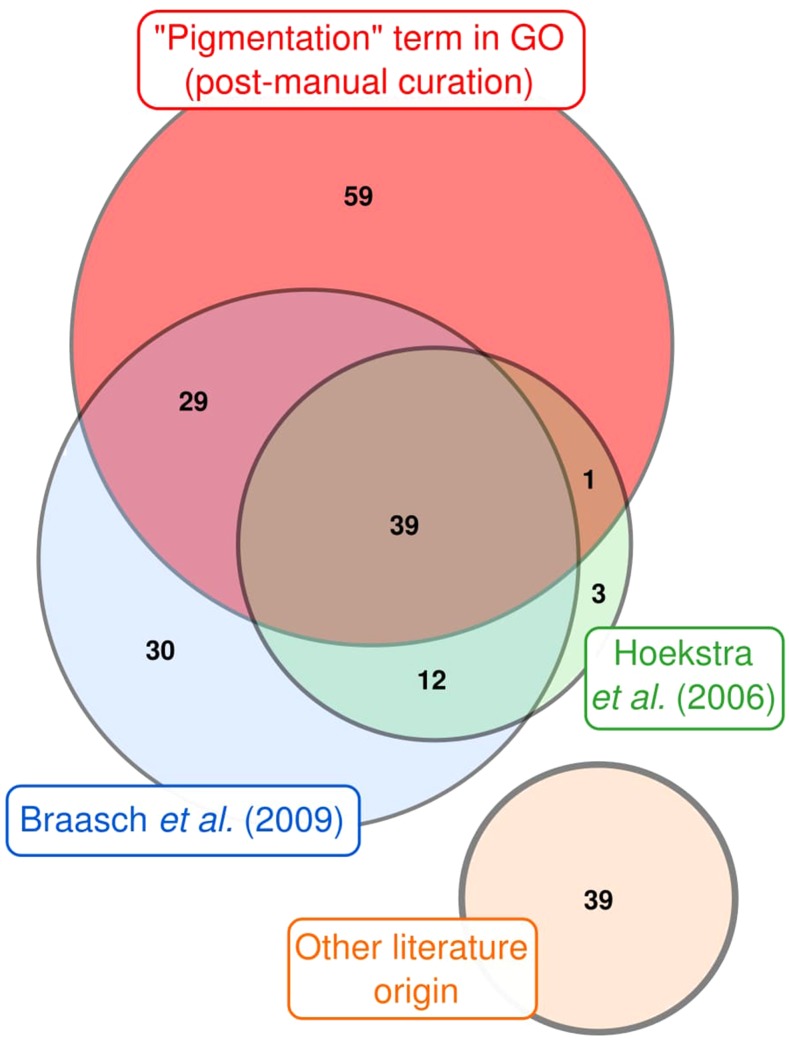
Origin of pigmentation genes studied in this work. Most PGs were either retrieved using the Gene Ontology term “pigmentation” (GO: 0043473) parsed for vertebrates (taxon id: 7742) or were previously cited in two studies (Hoekstra 2006; Braasch *et al.* 2009a). PGs described in more recent studies were also included (“other literature origin”). Numbers of genes are indicated. The Venn diagram was drawn using the BioVenn website (Hulsen *et al.* 2008).

**Figure 2:**
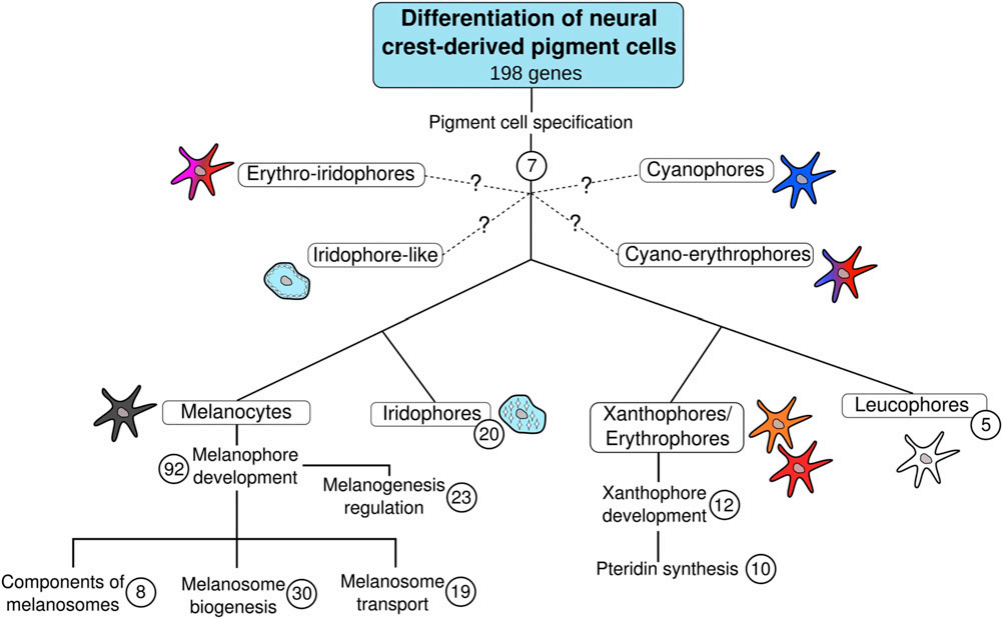
Classification of 198 vertebrate pigmentation genes according to functions and cell types. Numbers of pigmentation genes in (sub)categories are indicated in circles (some genes can be present simultaneously in different subcategories). Ontogenetic relationships between melanocytes, iridophores, xanthophores/erythrophores and leucophores are as proposed by (Kimura *et al.* 2014). Functional classification within melanocytes and xanthophores is adapted from (Braasch *et al.* 2009a). Question marks indicate that these newly described pigment cell types may originate from neural crest (NC), but that there is no experimental evidence for that yet.

To establish the PG dataset used in this study (Figure 1; Table S1), we first retrieved genes annotated under the Gene Ontology (GO) term “pigmentation” (GO: 0043473) in vertebrates (taxon identifier: 7742) (http://www.geneontology.org/, last accessed on March 08^th^, 2017). We manually verified the correspondence of automatic GO annotations with our definition of a pigmentation gene and, when necessary, excluded some genes from the list. For instance, *sod2* was not kept in our dataset because its deficiency causes red blood cell damage (Friedman *et al.* 2004). As such, it does not fit the definition above. In addition, all genes previously described in two former papers on vertebrate pigmentation were included (Hoekstra 2006; Braasch *et al.* 2009a). PGs from these different sources were sometimes redundant (Figure 1). Finally, we conducted a review of the literature to include PGs from more recent studies (Figure 1; Table S1). Based on previous work, pigmentation genes were classified into categories depending on their functions (Table S1; Figure 2) (Braasch *et al.* 2009a).

### Phylogenetic analysis

We define here as a *gene family* the genes that are derived by duplication and speciation from a pre-1R/2R ancestral deuterostome single-copy gene. After 1R/2R but before Ts3R, a gene family can be either multigenic if the ancestral gene has been kept as duplicates after 1R/2R, or monogenic if no duplicate has been maintained. We define as *pigmentation gene-containing family* (PGCF) a gene family containing at least one pigmentation gene. This implies that a PGCF can also contain genes that are not involved in pigmentation (experimental evidence), or for which no pigmentation function has been documented so far (lack of data) (see Figure S2 for a graphical representation of these definitions).

To assess the evolutionary history of each PGCF, and particularly how it was shaped by the four rounds of WGDs in vertebrates studied thereafter, sequences from 22 vertebrate genomes representing the major vertebrate lineages were analyzed. Eight sarcopterygian genomes including seven tetrapods were studied (*Latimeria chalumnae*, *Xenopus tropicalis*, *Anolis carolinensis*, *Gallus gallus* as well as the mammals *Ornithorhynchus anatinus*, *Monodelphis domestica*, *Mus musculus* and *Homo sapiens*). Thirteen actinopterygians (ray-finned fish) genomes were included in the study, encompassing a wide diversity of clades, including salmonids to study Ss4R (Near *et al.* 2012): the non-teleost spotted gar *Lepisosteus oculatus* (the closest outgroup to teleosts with sequenced genome that did not experience the Ts3R (Amores *et al.* 2011)) and the teleost species *Danio rerio*, *Astyanax mexicanus*, *Gadus morhua*, *Gasterosteus aculeatus*, *Tetraodon nigroviridis*, *Takifugu rubripes*, *Poecilia formosa*, *Xiphophorus maculatus*, *Oryzias latipes*, *Oreochromis niloticus*, *Oncorhynchus mykiss* and *Salmo salar*. To encompass the broad diversity of vertebrates we additionally included the genome of a chondrichthyan (cartilaginous fish), the elephant shark *Callorhinchus milii*. At the time of analysis, data from the lamprey genome were too incomplete to be included in the study. Sequences from five non-vertebrate deuterostomes that diverged from the vertebrate lineage before the 1R/2R WGDs were used as outgroups in the molecular phylogenies: the two urochordates *Ciona intestinalis* and *C. savignyi*, the cephalochordate *Branchiostoma floridae* (amphioxus) and the more distantly related ambulacrarians *Strongylocentrus purpuratus* (echinoderm) and *Saccoglossus kowalevskii* (hemichordate).

For each species, protein sequences of the longest isoform were retrieved from Ensembl v86 (www.ensembl.org; Oct. 2016) and NCBI (https://www.ncbi.nlm.nih.gov/, last accessed March 08^th^, 2017). When an Ensembl sequence was missing from the Ensembl annotation pipeline, we manually tested on the genome assembly (using Ensembl BLASTn method with the closest ortholog as query) whether this was due to gene loss or annotation skews. In the latter case, we used the FGENESH+ program of the Softberry suite (http://www.softberry.com/) to extract the gene sequence and predict the protein sequence.

Sequence alignments were performed using Clustal Omega v1.2.1 (Sievers and Higgins 2014) with default parameters and were individually manually curated for each PGCF (Blouin *et al.* 2009). Each alignment was then analyzed using the software prottest3 v.3.4.2 (Darriba *et al.* 2011) and the evolutionary model for tree-building was selected based on the Bayesian information criterion (Luo *et al.* 2010). Tree building was conducted with the best fitting model using the Maximum Likelihood method implemented in PhyML v3.1 (Guindon *et al.* 2010) with SH-aLRT support (Le and Gascuel 2008; Anisimova *et al.* 2011). The best-fitting model for each alignment is available (Table S2).

In order to assess if a duplication event was likely to be due to Ts3R and not to a small-scale duplication (SSD), and to identify large paralogons supporting Ts3R evidence, we conducted for each gene a synteny analysis using the Genomicus web browser (v86; http://genomicus.biologie.ens.fr/genomicus-86.01/cgi-bin/search.pl) (Louis *et al.* 2015).

Finally, as genes can be described in different organisms with different names, we used the unified nomenclature system for humans provided by the HGNC Database (Gray *et al.* 2015) to avoid nomenclature ambiguities.

### Retention rate calculation

There are two main methods to calculate retention rates (RRs) of duplicated copies after WGD (Brunet *et al.* 2017). Hereafter, the RR of duplicated copies was calculated as previously done by several authors (Figure S3) (Dehal and Boore 2005; Berthelot *et al.* 2014; Lien *et al.* 2016). When two ohnologs are present after one event of WGD, the duplicated copy has been maintained and its RR is 1. If only one copy is present after WGD, the duplicated copy has been lost and its RR is 0. We can calculate the average RR if several genes from a same family have undergone a same WGD (Figure S3).

### Pathway enrichment analysis and protein-protein interaction networks

For each gene (PGs and their corresponding vertebrate 1R/2R-ohnologs, if any), we retrieved the UniProt ID of the human ortholog in the UniProtKB/Swiss-Prot database (www.uniprot.org/, The UniProt Consortium 2015, last accessed March 08^th^, 2017). Gene IDs were used to test for enrichment in Gene Ontology (GO) terms with g:Profiler (http://biit.cs.ut.ee/gprofiler/) (Reimand *et al.* 2016) using default parameters and the human genome as background. The significance threshold for enrichment was set at 0.05 (corrected p-value for multiple testing with Benjamini-Hochberg FDR) and the “Hierarchical filtering” was set at “Best per parent group” to obtain only hierarchically highest GO terms. The number of interactions for each protein was obtained from the manually curated “Binary interactions” tab in UniProt.

### Data availability

All alignments and trees are available at FigShare; sequences were obtained from publicly available databases. All data necessary for confirming the conclusions presented in the article are represented fully within the article and its supplementary material. Supplemental material available at Figshare: https://doi.org/10.25387/g3.6061370.

## RESULTS

### Vertebrate pigmentation genes and pigmentation gene-containing families

We analyzed Gene Ontology databases and the existing literature to obtain a list of 198 vertebrate PGs that correspond to 181 PGCFs (the number of genes here is estimated after 1R/2R but before Ts3R; see Methods for details). Seventeen PGCFs contained two PGs, sometimes with different pigmentary functions (see Table S3). In contrast, most families (164/181) possessed only one PG, with its “non-PG” 1R/2R-ohnolog(s) (if any) having to the best of our knowledge no function described to date in the development of neural crest derived-pigment cells (either experimental evidence or absence of data).

The evolutionary history of nine gene families (*asip*, *foxd3*, *gja5*, *krt2a*, *rab8a*, *slc2a11*, *ugt1a8*, *xdh* and *zic2*) was too ambiguous to be included in our study because of the presence of many independent segmental duplication events and low phylogenetic resolution (this was previously reported for the *asip* gene family (Cal *et al.* 2017b); data not shown). We also excluded the *bcl2l11*, *defb103a* and *krtap21-1* families because these genes are either tetrapod-specific (*bcl2l11*) or eutherian-specific (*defb103a*, *krtap21-1)*. The final analyzed dataset therefore consisted of 184 PGs and 144 non-PG paralogs for a total of 328 total genes within 169 PGCFs.

After phylogenetic and genomic analyses of 27 deuterostomes species (including 22 vertebrates), orthology and paralogy relationships were established for all the 328 genes. A schematic representation of the occurrence of all genes with copy numbers (corresponding to a total of 9,112 sequences) is provided for each studied species (see Figure S4A-J and an example case Figure S5).

### Retention rates of pigmentation genes after the fish-specific WGDs (Ts3R and Ss4R) are higher than genome average

**Figure 3:**
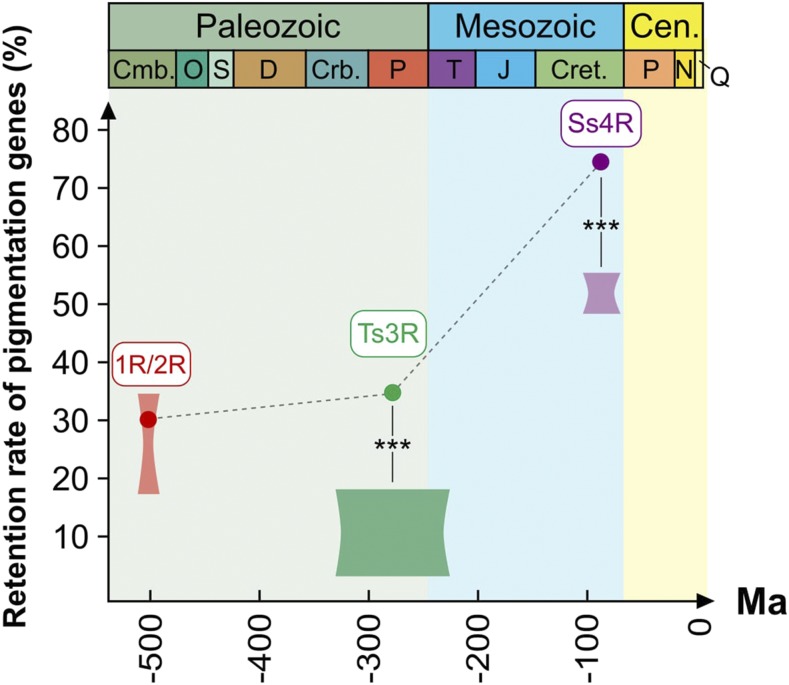
Retention rates for the whole pigmentation gene repertoire after successive WGDs in vertebrates. At each WGD event, heights of shaded areas represent the genome-wide retention rates estimates based on multiple studies (see text for detail); widths of shaded areas represent the temporal estimates available in the literature for each WGD event. Dots represent the retention rate for each WGD event when considering only PGs. Pigmentation repertoire retention rate is higher than genomic average after Ts3R- and Ss4R-WGDs. ***: *P* < 10^−7^ (chi2 test, see text for values).

We first observed that the global retention rate (RR) of PGs after Ts3R was 34.2% (= 63/184) (Figure 3; Table 1). Interestingly, this rate was significantly higher than the range of values reported for the whole genome, *i.e.*, 4–18% depending on the datasets used (Jaillon *et al.* 2004; Woods *et al.* 2005; Brunet *et al.* 2006; Howe *et al.* 2013; Inoue *et al.* 2015) (*P* = 1.8 × 10^−8^, chi2 test). Of note, values published in earlier studies were recalculated if needed before comparison to be consistent with our calculation method. This indicated that PGs have been globally more frequently retained as duplicates than other genes after the Ts3R WGD.

**Table 1:** Retention rates after Ts3R and Ss4R of genes present in pigmentation gene-containing families

WGD event	Overall PG retention rate	PGs in monogenic PGCFs	PGs in multigenic PGCFs	Non-PGs in multigenic PGCFs
Ts3R	34.2% (63/184)	16.9% (13/77)	46.7% (50/107)	37.1% (52/140)
Ss4R	75.2% (182/242)	72.2% (65/90)	73.2% (112/153)	71.5% (133/186)

After the salmon-specific Ss4R WGD, the RR was 75.2% for PGs (182/242; Figure 3; Table 1). Again, this value was higher than the value reported for the whole genome (48–55%, *P* = 4.3 × 10^−10^, respectively, chi2 test) (Berthelot *et al.* 2014; Lien *et al.* 2016). Hence, PGs have also been preferentially retained after the Ss4R duplication compared to other genes.

We eventually assessed whether the retention rates after Ts3R and Ss4R would differ between genes from different functional categories in pigmentation (see Figure 2). There was no significant group effect after both Ts3R and Ss4R (p-value of Wald test = 0.30 and 0.34, respectively) (Figure S6A and 6B).

### Pre-1R/2R pigmentation genes ancestors are not preferentially retained after 1R/2R

The ancestral vertebrate double event of WGD (1R/2R) is assumed to have played a major role in the evolution of vertebrate gene families (Dehal and Boore 2005; Xie *et al.* 2016). We therefore investigated the consequences of 1R/2R on PG evolution (note that the respective effects of 1R *vs.* 2R cannot be differentiated particularly due to the relatively short period of evolution between these two ancient WGD events). However, whether the ancestral pre-1R/2R genes had a function or not in pigmentation is not documented, hence the RR after 1R/2R could not be calculated for PGs *per se*. Instead, we calculated the post-1R/2R retention rates for pre-1R/2R pigmentation gene ancestors. We obtained a value of 30.4% (average RR for 169 PGCFs), which is well in the range of those previously reported for the complete genome (17.0–34.5% ; (Dehal and Boore 2005; Xie *et al.* 2016) (Figure 3).

**Figure 4:**
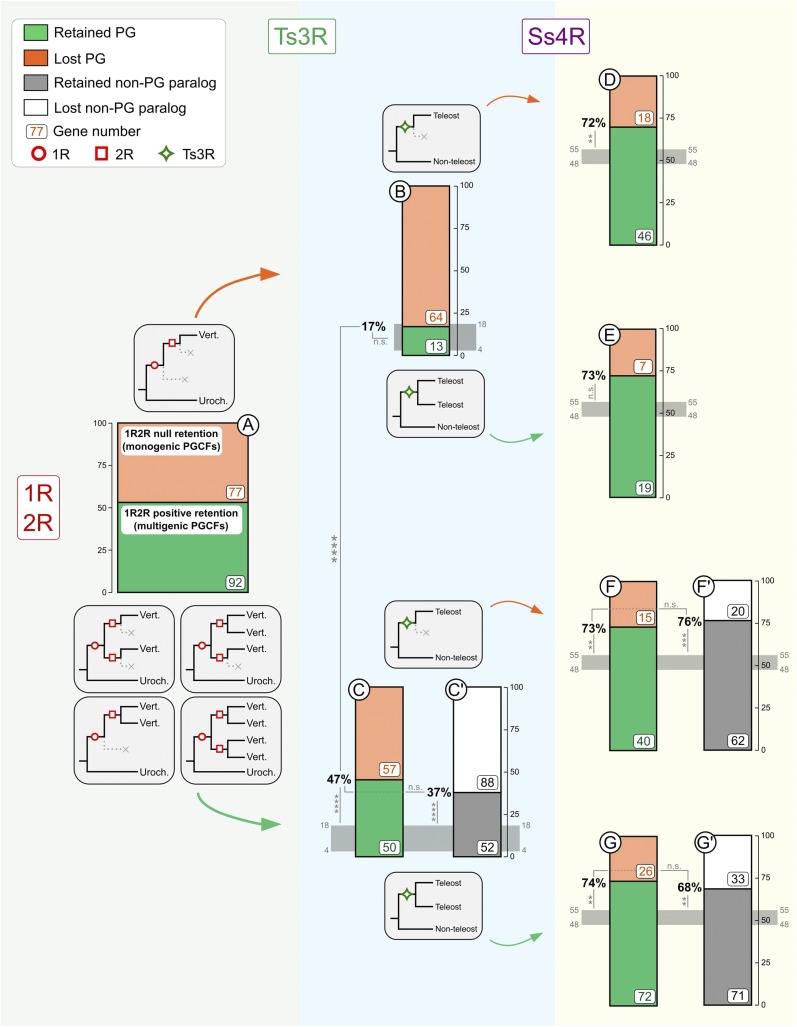
Retention rates for pigmentation genes from monogenic and multigenic families after successive WGDs in vertebrates. Green and orange boxes (respectively black and white) represent number of genes that are whether retained or not at the considered WGD for PGs (respectively non-PGs paralogs in multigenic PGCFs). Total numbers of genes per dataset are indicated. For 1R/2R, the bar does not represent retention rates but the 77 monogenic PGCFs *vs.* the 92 multigenic PGCFs. Retention rates are indicated (between 0 and 100%) for Ts3R and Ss4R. Horizontal gray bands represent the genome average retention rate observed in the literature for Ts3R (4–18%) and Ss4R (48–55%). *: *P* < 0.05; **: *P* < 0.01; ***: *P* < 0.001; ****: *P* < 0.0001; n.s.: not significant at a 0.05 level (chi2 test).

If at least two post-1R/2R ohnologs are retained, the PGCF is “multigenic”. Instead, if there is only one retained gene, the family is “monogenic” and only contains the pigmentation gene without any ohnolog. About half of PGCFs (92/169) (54.4%) were multigenic (Figure 4, case A). In these 92 multigenic families, 107 PGs were found (15 families harbored two PGs; Table S3) as well as 144 non-PGs. In contrast, 45.6% (77/169) of PGCFs were monogenic. When comparing the retention rates for the different genes in functional pigmentation categories (Figure 2), we found that the “melanosome biogenesis” group was enriched in monogenic PGCFs and therefore had a lower RR after 1R/2R compared to other groups (p-value of Wald test = 0.015) (Figure S6C).

### Only PGs from 1R/2R multigenic families have higher retention rates after Ts3R compared to genomic average

We then assessed whether the pigmentation genes in post-1R/2R multigenic families had more chance to be retained as duplicates also after Ts3R than post-1R/2R singletons. While the overall RR for PGs was 34.2% after Ts3R (see above), we showed that genes in multigenic PGCFs had a higher retention rate after Ts3R (50/107 = 46.7%; Table 1; Figure 4, case C; *P* = 2.6 × 10^−5^, chi2 test) than PGs in monogenic PGCFs (13/77 = 16.9%; Table 1; Figure 4, case B). After Ts3R, the RR of PGs in monogenic 1R/2R PGCFs was within the reported genome range of retention (Figure 4, case B) while Ts3R RR for PGs in multigenic 1R/2R PGCFs was significantly higher (Figure 4, case C; *P* = 2.4 × 10^−14^, chi2 test). Thus, the global high RR for PGs after Ts3R is principally due to the high RR of PGs from 1R/2R multigenic families.

### PGs from both monogenic and multigenic 1R/2R PGCFs have similar high retention rates after Ss4R

We subsequently compared the RR after Ss4R of PGs from 1R/2R monogenic *vs.* multigenic gene families. We showed that PGs that were in monogenic 1R/2R PGCFs (Table 1; Figure 4, cases D and E) had a RR higher than average similar to that of PGs in multigenic PGCFs (Table 1; Figure 4, cases F and G): [19+46]/[26+64] = 72.2% *vs.* [40+72]/[55+98] = 73.2%, respectively (*P* = 0.98, chi2 test). Thus, not only PGs from multigenic 1R/2R PGCFs are preferentially retained after Ss4R - as observed for Ts3R - but also PGs from 1R/2R monogenic families - in contrast to Ts3R.

Additionally, we assessed a possible correlation between Ts3R and Ss4r RRs for PGs in PGCFs. We showed that genes retained after Ts3R (Figure 4, cases D and F) were not more retained after Ss4R than genes not retained after Ts3R (Figure 4, cases E and G): [46+40]/[64+55] = 72.3% *vs.* [19+72]/[26+98] = 73.4%, respectively (*P* = 0.96, chi2 test). However, both have a higher RR than genomic average. Therefore, PGs have been retained at a higher rate than genomic average after Ss4R independently from the copy number of their gene family after both 1R/2R and Ts3R.

### PGs and their non-pigmentary paralogs have similar high RRs after teleost-specific WGDs

We have then compared within 1R/2R multigenic gene families the RR of PGs with that of their non-pigmentary paralogs after both Ts3R and Ss4R. We first showed that the retention rate after Ts3R for non-pigmentary paralogs (37.1% = 52/140; Table 1; Figure 4, case C’; Table 1) was as high as the retention rates for PGs in general (34.2% = 63/184; *P* = 0.67, chi2 test) and particularly for PGs in multigenic PGCFs (46.7% = 50/107; Table 1; Figure 4, case C; *P* = 0.17, chi2 test). This rate was higher than the range of values reported for the whole genome (*P* = 6.2 × 10^−9^, chi2 test). Thus, PGs and their corresponding paralogs in multigenic PGCFs are both highly retained after Ts3R.

After Ss4R, as previously stated, PGs were retained at 73.2% (Table 1; Figure 4, cases F and G). Similarly, their paralogs were retained at [62+71]/[82+104] = 71.5% (Table 1; Figure 4, cases F’ and G’). The values obtained were both higher than the global genomic range of retention (Figure 4, cases F’ and G’). Hence, PGs and their paralogs in multigenic PGCFs are equally retained after both Ts3R and Ss4R.

### Genes in multigenic PGCFs might be at the core of protein-protein interaction networks

Previous studies have shown that genes that are sensitive to dosage balance and those encoding proteins with a high number of interactions are more likely to be retained after WGD events (Freeling and Thomas 2006; McGrath *et al.* 2014; Bright *et al.* 2017). Hence, we hypothesized that genes in families with high RRs would have more “central” molecular functions (*e.g.*, transcription factors or intracellular signaling actors such as protein kinases) than PGs in families with low retention rates. Accordingly, enrichment analyses in Gene Ontology terms on genes in multigenic PGCFs showed that the most significant “Molecular Function” term was “transcription regulatory region DNA binding” (Table S4A). In contrast, PGs in monogenic PGCFs were mostly enriched in metabolic enzymes (Table S4B).

In addition, we estimated the number of interaction partners for each pigmentation protein using the UniProtKB/Swiss-Prot database (self-interactions being excluded). We showed that products of genes in multigenic PGCFs held more interactions on average than PGs in monogenic PGCFs (n = 5.5 *vs.* n = 2.2 on average, respectively; p-value of Welch *t*-test = 0.003). PGs and non-PGs in multigenic PGCFs, which have similar retention rates, also display similar average interaction number (n = 7.6 and n = 3.8, respectively; p-value of Welch *t*-test = 0.08).

## DISCUSSION

In this study, we first showed that pigmentation genes were on average more retained than the genome average after both teleost-specific Ts3R and Ss4R WGDs, but not after the 1R/2R WGDs. Non-pigmentary post-1R/2R ohnologs of pigmentation genes are also more retained than the rest of the genome after WGDs in teleosts, suggesting that the function in pigmentation is not the only factor determining gene retention. After the evolutionary ancient 1R/2R and Ts3R WGDs, specific categories of genes have been repeatedly preferentially retained, which might have more central molecular functions compared to genes in monogenic PGCFs.

### Pigmentation genes and retention rates

Pigmentation is a trait determined by many molecular actors and a strict definition of a “pigmentation gene” seems impossible. In particular, genes that are involved in the patterning of pigmentation such as *lvrn*/*taqpep* are not necessarily involved in the development of chromatophores, but rather influence the development of surrounding cell territories (Kaelin *et al.* 2012). In this work, we have studied genes that are implicated in the development of pigment cells as defined by (Schartl *et al.* 2016), *i.e.*, cells whose embryonic origin is the neural crest. Pigment cells of the retinal pigment epithelium, as they are not involved in body coloration and derive from the optic cup - a neuroepithelium derivative - were not studied here. We ascertained that genes qualified as “non-pigmentary paralogs” were not implicated in pigmentation by examining the annotations of the available mutants for these genes in the Mouse Genome Informatics (http://www.informatics.jax.org/allele) as well as in the Zebrafish Information network databases (last accessed February 9^th^, 2018), two of the most comprehensive mutant databases in vertebrates. Most presumed “non-pigmentary” post-1R/2R paralogs of PGs had indeed no “pigmentation” mutant described, confirming the robustness of the dichotomization “pigmentation”/“non-pigmentation” used here (data not shown). The lack of functional evidence can nevertheless either reflect a “real” absence of effect on pigmentation, or an artifactual lack of evidence due to a small number of studies.

### A putative ancestral pigmentation gene repertoire in jawed vertebrates

Mammals only have one pigment cell type (melanocytes), while birds, lepidosaurians and amphibians have four to five. As already suggested by others, early gnathostomes (jawed vertebrates) were likely to possess most of these pigment cell types (Lapedriza *et al.* 2014). This implies that secondary losses of pigment cell lineages occurred in mammals, while new pigment cell types – *e.g.*, cyanophores – arose only in teleosts, with an important diversity of pigmentation patterns (Bagnara *et al.* 2007). In addition to the diversity in terms of pigmentation patterns, teleost fish also have the highest number of pigment cell types in vertebrates.

Our phylogenomic analysis of 198 PGs and their corresponding families confirmed the ancestry of the genetic control of the melanocyte/melanophore lineage in jawed vertebrates. This is supported by the conservation of many genes such as *mitf*, *mc1r*, *sox10*, *kit/kitlg* as well as genes from the Wnt pathway in both sarcopterygians and actinopterygians (Rawls *et al.* 2001; Braasch *et al.* 2009a) (Figure S7). The information on the functional conservation of underlying genes implicated in the development of other pigment cells absent from mammals is scarcer in the available literature. However, we have shown that genes involved in the formation of leucophores, erythrophores/xanthophores and iridophores are present in different vertebrate lineages, here again suggesting common ancestry (see corresponding PGCFs in Table S1 and Figure S4). These include the iridophore marker *foxd3* and the leucophore and xantho-/erythrophore genes *slc2a11* and *slc2a15* (Curran *et al.* 2010; Kimura *et al.* 2014). As previously shown by others, the xanthophore marker *somatolactin* (*smtl*) is present in actinopterygians and in the coelacanth but not in other tetrapods, suggesting a secondary loss in this lineage (Jiang *et al.* 2011).

Mutations of some pigmentation genes in non-mammalian species affect more pigment cell types than the sole melanocytes. This suggests that there are close ontogenetic relationships between pigment cell types. For instance, the transcription factor Mitf, which is involved in melanocyte formation in mammals, interacts with Foxd3 to determine the development of melanocytes and iridophores in zebrafish (Curran *et al.* 2010). In zebrafish and medaka, evidence of common progenitors has been found for leucophores and xanthophores on the one hand, and for iridophores and melanocytes on the other hand (Curran *et al.* 2010; Kimura *et al.* 2014). More functional studies are needed in other lineages including amphibians or lepidosaurians to assess the ancestry of ontogenetic relationships between pigment cell types in jawed vertebrates.

The presence of ancestral genetic networks driving the formation of pigment cell lineages early in vertebrate evolution is strongly supported by the conservation of pigmentary functions for genes in different vertebrate lineages. For instance, the *asip* locus has a conserved role in dorso-ventral pigment asymmetry in vertebrates, which results from a switch of melanin type in mammals and from the differential distribution of several types of chromatophores – including melanocytes – in both spotted gar and zebrafish, so presumably in the ancestor of ray-finned fish (Ceinos *et al.* 2015; Cal *et al.* 2017a). *Sox10* mutants present defects in melanocytes in mammals and in all pigment cell types in zebrafish (the corresponding mutant was incidentally coined *colorless*), suggesting a central function in the development of these neural crest derivatives (Rawls *et al.* 2001). Thus, the results provided here on the phylogenetic distribution within vertebrates of genes involved in pigmentation may provide a framework for the evolutionary and developmental understanding of the underlying genetic basis of vertebrate (and teleost) pigmentation.

### Successive rounds of WGDs have extended the vertebrate pigmentation gene repertoire especially in teleost fish

While two first rounds of WGDs (1R/2R) have occurred early during the course of vertebrate evolution around 500 million years ago (Mya) (Putnam *et al.* 2008), a 3^rd^ round of WGD specific to ray-finned fish (Ts3R) has taken place between 225 and 333 Mya before the teleost radiation (Hurley *et al.* 2007; Santini *et al.* 2009; Near *et al.* 2012). A 4^th^ round of WGD (Ss4R) occurred in the salmonid fish lineage about 80-100 Mya (Macqueen and Johnston 2014; Berthelot *et al.* 2014; Lien *et al.* 2016). Such WGDs are drastic events leading to the doubling of genomes, which can strongly contribute to evolution at both gene and organismal levels (Conrad and Antonarakis 2007). As already suggested for Ts3R (Braasch *et al.* 2009a), we show that these WGDs have increased the evolvability of the pigmentation gene repertoire and therefore have contributed to the diversification of pigmentation cell types and patterns in vertebrates. This phenomenon might be especially true for teleost fish: for Ts3R and Ss4R, the retention rates obtained for the pigmentation repertoire are higher than genomic average, respectively), while they are in the genome-wide average for 1R/2R (Figure 3). The high pigmentation diversity observed in teleost fish in general, both in terms of chromatophore types and pigment patterns, is thus correlated to an extension of the repertoire of PGs after WGDs.

In salmonids, to the best of our knowledge, there is no obvious link between the Ss4R duplication and higher chromatophore diversity compared to other teleosts. This might be explained by the observed time-lag between WGD and evolutionary diversification in this family (Robertson *et al.* 2017) (but see (Colihueque 2010) for a discussion on the diversity of salmonid pigmentation). Of note, more salmonid genomes must be analyzed to avoid bias in the calculation of retention rates.

### Pigmentation genes are predestinated to either loss or retention after ancient WGDs

After analyzing the long-term evolution of pigmentation genes after ancient WGDs, we observed that those that have been already retained after 1R/2R are also preferentially kept as duplicates after Ts3R. This indicated that after successive WGDs and long-term evolution, some pigmentation gene duplicates are recurrently retained, while others are preferentially lost. However, this phenomenon was not observed for the salmonid-specific Ss4R, after which PGs were retained at similar higher rates independently from their retention rate after previous WGDs. This is consistent with what has been previously found in salmon at the genome-wide level (Lien *et al.* 2016) but not in the rainbow trout (Berthelot *et al.* 2014). A potential reason to explain this apparent discrepancy could be the relatively recent occurrence of this event (ca. 96 Mya), with gene loss associated with the rediploidization process being still ongoing, at least in the Atlantic salmon (Robertson *et al.* 2017).

While general factors governing the destiny of genes after duplication events are still being debated (Rogozin 2014), several drivers of high retention after WGDs have been already described. Genes encoding transcription factors, signal transduction proteins and other proteins implicated in developmental processes are usually retained in excess compared to other genes (Conant and Wolfe 2008). This is not only valid for teleost fish (Steinke *et al.* 2006; Brunet *et al.* 2006; Hufton *et al.* 2008; Kassahn *et al.* 2009; Berthelot *et al.* 2014) but also for other vertebrates (Blomme *et al.* 2006; Session *et al.* 2016), yeast (Davis and Petrov 2005; Guan *et al.* 2007), plants (Seoighe and Gehring 2004; Maere *et al.* 2005; Jiang *et al.* 2013; Li *et al.* 2016) and paramecium (McGrath *et al.* 2014). At a protein family level, this is the case for receptor tyrosine kinase genes, which have been more frequently retained than other genes after 1R/2R and Ts3R (Brunet *et al.* 2016). The retention for transcription factor genes of the *Sox* family is also higher than genome average after Ts3R (Voldoire *et al.* 2017). Accordingly, we showed that pigmentation genes retained as duplicates after 1R/2R were enriched in transcription factor activity (Table S4A) compared to pigmentation genes in monogenic PGCFs (Table S4B).

It has also been proposed in the “dosage balance hypothesis”, since all gene product quantities are doubled after a WGD, that the loss of one gene copy would be deleterious, especially if the protein has a central role in the cell with many interactors (Lemos *et al.* 2004; Birchler and Veitia 2007). As such, ohnologs are more often conserved than segmental duplicates because the latter immediately disrupt dosage balance (Makino and McLysaght 2010). In addition, at a genome-wide scale, duplicated genes tend to have more interactions than singleton genes (He and Zhang 2005) and proteins encoded by retained WGD ohnologs have significantly more protein interactions than proteins encoded by genes that are not duplicated (Wu and Qi 2010). Accordingly, we found that proteins encoded by PGs and in multigenic families held more interactions on average than PGs in monogenic families.

Finally, recurrent differential retention rates might be linked to pigmentation functions with different degrees of evolutionary constraints and evolvability: some genes involved in more conserved functions might be more refractory to retention than others. In our analysis, genes involved in “melanosome biogenesis” showed a lower retention rate after 1R/2R. In addition, although not significant, all genes involved in “pigment cell differentiation” (see Figure 2) belonged to multigenic PGCFs (Figure S6C), suggesting that genes that are involved in the cell differentiation process are highly retained.

### Non-pigmentary paralogs of pigmentation genes also have high retention rates after teleost WGDs

We have reported here that non-pigmentary paralogs have similar high retention rates than the PGs from the same family. This was observed in multigenic PGCFs not only after the ancestral teleost-specific Ts3R but also after the more recent salmonid-specific Ss4R. This could suggest that the function in pigmentation is not the unique, or even not the main driver of high retention rates in PGCFs. As discussed above, the molecular nature and biochemical properties of a protein are certainly major factors determining the retention rate of a gene after genome duplication. This would explain why paralogous genes from a same gene family, which encode similar proteins, have similar RR independently from their biological function (involvement in pigmentation or not). However, one might expect, as observed for 1R/2R and Ts3R (see above), that a given gene family encoding related proteins will display a conserved bias of retention *vs.* loss after different WGDs. This is not the case after Ss4R, since PGs and their paralogs have been equally highly retained independently from their RR after the more ancient 1R/2R and Ts3R WGDs.

On the other hand, one might also consider that our category “non-pigmentation genes” might include PGs. Some post-1R/2R non-PG genes have been classified as such due to the absence of functional data, some genes might indeed play so far an unknown role in pigmentation. In addition, our functional annotation is mostly (but not exclusively) based on data from mouse mutants. It appears largely possible that some mammalian “non-PG genes” might have a pigmentation function in the ray-finned fish lineage. Such cases have been already documented, including the colony-stimulating factor 1 receptor gene *csf1ra* (aka *fms*) (Parichy *et al.* 2000) and the *erbb3b* gene, which encodes an EGFR-like receptor tyrosine kinase (Budi *et al.* 2008). Interestingly, both genes have been duplicated by the 1R/2R and 3R WGDs, and *csf1r* is a 1R/2R ohnolog of the well-studied pigmentation gene *kit* (Brunet *et al.* 2016). Such functional differences might be explained by a loss of function in pigmentation in the tetrapod/mammalian lineage, or alternatively by a gain of pigmentary function in the ray-finned/teleost fish lineage, as observed for several transcriptional regulators during the evolution of neural crest at the basis of vertebrates (Meulemans and Bronner-Fraser 2005). The activity of a transcription factor has indeed already been shown to be co-opted to control novel pigmentation patterns in fly, with the evolution of specific binding sites in a *cis*-regulatory element (Gompel *et al.* 2005). The recruitment to pigmentation of genes involved in other processes such as organ development has mainly been shown in arthropods but is likely to occur in vertebrates too (Werner *et al.* 2010; Monteiro 2012). In teleost fish, *fhl2b*, a gene duplicated after Ts3R, has been shown to be involved in the development of iridophores (more specifically in the formation of egg-spots in cichlids) consecutively to the insertion of a transposable element that induced changes in the regulation of its expression (Santos *et al.* 2014).

Finally, after a duplication event, gene conversion can “homogenize” the sequences of the two duplicates and thus counteracts duplicate loss through a pseudogenization process. This process can also contribute to their diversity, as reported for some gene families including cadherins (Noonan 2004; Ohta 2010; McGrath *et al.* 2014). Thus, even if they are not directly involved in pigmentation, the presence of non-pigmentary paralogous copies might favor the maintenance and evolution of pigmentation genes through gene conversion and ectopic recombination.

### Conclusion

The present analysis supports a major role of WGDs in the diversification of the pigmentation gene repertoire, especially in the teleost fish lineage. This might have promoted the diversity of pigment cell lineages observed in these animals compared to other vertebrates. Besides, the functional basis of pigmentation is still being uncovered in non-mammal vertebrate lineages that harbor a high chromatophore diversity, including amphibians, lepidosaurians and birds. Thus, our genome-wide characterization of 1R/2R pigmentation gene-containing families provides an important framework to approach the ancestral pigmentation gene repertoire in vertebrates and better understand the evolution of pigmentation in relation to the gain or loss of specific pigment cell types.
